# Mechanistic Insights into Mercury Photoreduction: Effects of Dissolved Organic Matter and Inorganic Carbon in Seawater

**DOI:** 10.1021/acs.est.5c15961

**Published:** 2026-03-27

**Authors:** Sangwoo Eom, Huu-Viet Nguyen, Asif Qureshi, Seunghee Han

**Affiliations:** † Department of Environment and Energy Engineering, 65419 Gwangju Institute of Science and Technology (GIST), Gwangju 61005, Republic of Korea; ‡ Department of Climate Change and Department of Civil Engineering, 233600 Indian Institute of Technology (IIT) Hyderabad, Sangareddy, Telangana 502285, India

**Keywords:** dissolved gaseous mercury, evasion, indirect photolysis, thiols, bicarbonate, aromatic organic matter, reactive oxygen species, two-step reversible kinetic model

## Abstract

Atmospheric mercury (Hg) is primarily derived from marine evasion of Hg(0) produced through the photoreduction of Hg­(II) in seawater. Although the mechanisms of Hg­(II) photoreduction in seawater have been extensively investigated, the coupled effects of dissolved inorganic carbon and specific dissolved organic matter (DOM) structures remain unresolved. Here, we report the pseudo-first-order rate constants (*k*_r_) for the gross photoreduction of Hg­(II) in the presence of thiols in deionized water (DIW), phosphate buffer solution (PBS), and artificial seawater (ASW) under UV-A irradiation. For aliphatic thiols, *k*_r_ was below the detection limit in DIW and PBS but increased to 0.081–0.50 h^–1^ in ASW, exhibiting a concentration-dependent trend. In contrast, aromatic thiols yielded *k*_r_ up to 1.5 h^–1^ in DIW, PBS, and ASW, with no evident concentration dependence. In the absence of thiols, aromatic DOM decreased *k*_r_ possibly by reoxidizing Hg­(I) through the production of intermediate oxidants. Notably, *k*_r_ values for Hg–glutathione in PBS containing NaHCO_3_ were comparable to those in natural seawater, whereas other sea salts produced *k*_r_ below detection limits, likely because HCO_3_^–^ and CO_3_^2–^ scavenge Cl^•^ and Cl_2_^•–^, thereby enhancing the Hg­(II) reduction rate. Overall, a two-step reversible Hg­(II) reduction model involving the Hg­(I) intermediate is proposed to account for the dynamic redox equilibrium of Hg, underscoring the critical roles of aliphatic thiolic DOM, nonthiolic aromatic DOM, chloride, and bicarbonate in modulating *k*_r_ variability in seawater.

## Graphical abstract

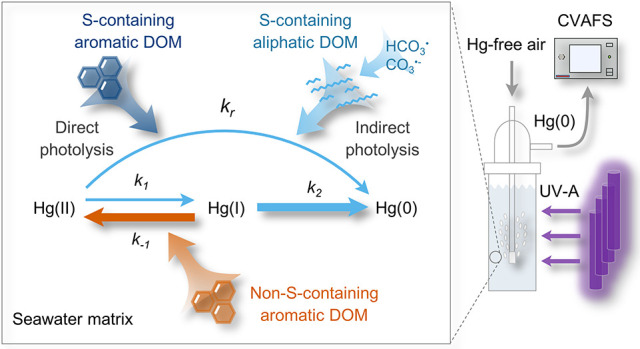

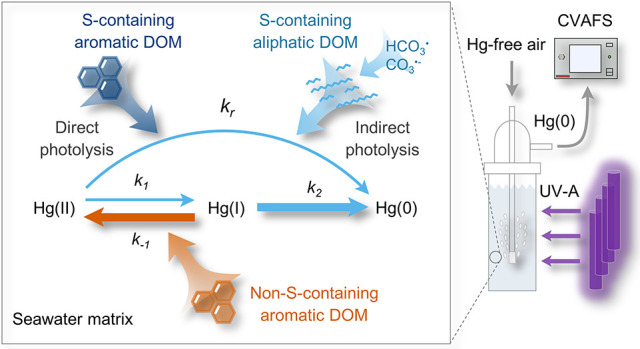

## 1 Introduction

Mercury (Hg) is a global pollutant capable of long-range atmospheric transport from low-latitude regions to the polar areas in the form of gaseous elemental mercury (GEM).^1,2^ Once deposited onto the surface ocean, GEM and its oxidized form, Hg­(II), undergo a series of biological and chemical transformations that can produce methylmercury (MeHg)—a highly toxic and bioaccumulative Hg species. The formation of dissolved gaseous mercury (DGM), primarily present as Hg(0), through the reduction of Hg­(II) in surface seawater, decreases the pool of bioavailable Hg­(II) for microbial methylation while simultaneously enhancing atmospheric GEM concentrations via evasion.^3,4^ Currently, DGM evasion from the ocean surface accounts for approximately 60% of the total Hg flux to the global atmosphere.^2^ Although DGM can form through both abiotic photoreduction and biotic dark reduction in surface seawater, uncertainties in these reaction rates contribute substantially to variability in modeled atmospheric Hg concentrations and deposition fluxes.^5^ Despite their importance, the mechanisms controlling biotic and abiotic Hg­(II) reduction remain poorly understood, and only a limited number of studies have examined how seawater chemistry influences these reaction rates.^3,4^

The rate constants for Hg­(II) photoreduction in surface ocean waters are one to 2 orders of magnitude higher than those for biotic Hg­(II) reduction, suggesting that the evasion flux of Hg(0) from the ocean surface to the atmosphere is predominantly driven by photoreduction in the sunlit mixed layer.^3,6^ The rate constants for Hg(0) oxidation have been characterized for various oxidants, including hydroxyl and carbonate radicals, as well as thiol-containing dissolved organic matter (DOM).^7,8^ In contrast, parametrizations of Hg­(II) photoreduction remain largely limited to light intensity–scaled values.^5,9^ A suppressive effect of chloride on Hg­(II) photoreduction has been reported in both laboratory and field studies. For example, laboratory experiments with estuarine water showed that the Hg­(II) photoreduction rate constant (*k*_r_) decreased at higher salinities (>13.5 g L^–1^), corresponding to an increased fraction of Hg–Cl complexes.^4^ Similarly, elevated Cl^–^ concentrations (>0.1 M) in aqueous solutions have been shown to reduce the Hg­(II) photoreduction rate due to enhanced oxidation of Hg(0) and Hg­(I).^10,11^ However, variations in *k*_r_ observed in natural seawater (0.24–0.46 h^–1^) were not linearly correlated with salinity within the range of 26–32 practical salinity unit (PSU) in the marginal seas of the North Pacific Ocean, indicating that *k*_r_ may also be influenced by DOM and other major ions in seawater, in addition to chloride.^3^

Photoreduction of Hg­(II) can proceed via ligand-to-metal charge transfer (LMCT; i.e., direct photoreduction)^12,13^ or be mediated by photochemically produced reactive intermediates that transfer electrons to Hg–DOM complexes (i.e., indirect photoreduction).^14–16^ Under the low Hg-to-dissolved organic carbon (DOC) ratios characteristic of natural seawater (<4 nmol mg^–1^),^17^ Hg­(II) likely binds to thiol (−SR) functional groups in DOM, forming Hg­(SR) and Hg­(SR)_2_ complexes that serve as photoreduction substrates. Photoreduction of Hg­(II) complexed with alkanethiols (C_3_–C_5_) in freshwater has been reported to occur through both direct and indirect photolysis, with rate constants ranging from 2.9 × 10^–4^ to 7.2 × 10^–4^ h^–1^ under UV irradiation.^13^ These values are substantially lower than those observed in seawater under UV–A or UV–B exposure (0.15–0.93 h^–1^).^3,4,6^ The higher *k*_r_ of Hg­(II) in seawater may result from the formation of highly photoactive Hg­(II)–DOM complexes that facilitate direct photoreduction and/or from enhanced indirect photolysis associated with sea salt components.^12,16^ Aromatic DOM exhibits relatively high photooxidation rate constants because it generates diverse intermediate oxidants such as hydroxyl radicals (^•^OH), singlet oxygen (^1^O_2_), excited triplet-state DOM (^3^DOM*), and carbonate radicals (CO_3_^•–^),^7,18^ whose steady-state concentrations generally increase with salinity.^19^ In addition, aromatic DOM can enhance Hg­(II) photoreduction by producing intermediate reductants such as organic free radicals and superoxide radicals (O_2_^•–^).^15,20^ The superoxide radical, O_2_^•–^, has been identified as a key species facilitating photoreduction, as its dismutation proceeds slowly in seawater due to stabilization by sea salt cations.^21^ In summary, both inorganic salts and DOM regulate the rate of Hg­(II) photoreduction in seawater by influencing the formation and persistence of reactive intermediate species.

Hg­(II) photoreduction in aqueous systems can proceed either through a two-step, one-electron transfer pathway—where Hg­(II) is sequentially reduced to Hg­(I) and then to Hg(0)—or via a direct two-electron transfer from Hg­(II) to Hg(0).^22,23^ Most experimental studies and global models have parametrized Hg­(II) photoreduction assuming a two-electron transfer mechanism.^3,4,6,9^ However, recent evidence suggests that the direct two-electron reduction of Hg­(II) to Hg(0) is an energetically unfavorable and relatively slow process, whereas a two-step, one-electron transfer pathway is more plausible.^24^ This sequential mechanism may be mediated by thiyl radicals (RS^•^), as radical pairs of RS^•^ and Hg–thiyl radicals [^•^Hg­(SR)] can be produced from Hg­(SR)_2_ complexes under UV irradiation.^23^ Despite its potential significance, few studies have quantitatively characterized the rate constants for the individual steps of the one-electron transfer pathway, particularly under varying DOM and sea salt compositions.

This study investigates how seawater chemistry—specifically aliphatic and aromatic thiolic DOM, nonthiolic aromatic DOM, and sea salts—influences the kinetics and mechanisms of Hg­(II) photoreduction under UV-A irradiation. To this end, pseudo-first-order *k*_r_ values were determined for systems containing Hg­(II) and varying DOM concentrations in deionized water (DIW), 1 mM phosphate buffer solution (PBS) at pH 8, and artificial seawater (ASW) to separate the effects of seawater pH and ionic composition on *k*_r_. Using the experimentally derived kinetic data, key seawater constituents governing the *k*_r_ of Hg­(II) were identified. Furthermore, the reaction kinetics were simulated using consecutive rate constants obtained from a two-step reversible Hg­(II) reduction model. This integrated approach enables identification of the seawater components most critical in regulating Hg­(II) photoreduction and elucidation of the dominant reaction pathways operating in artificial and natural seawater.

## 2 Materials and Methods

### 2.1 Photoreduction Setup

The Hg­(II) photoreduction experiments were conducted using a custom-built photoreactor that accommodates up to eight UV lamps and maintains a constant temperature using an internal circulation system. Seven hundred mL of reaction matrix (DIW, 1.0 mM PBS, or ASW without Hg­(II) and DOM) was first purged with Hg-free air (∼1.2 L min^–1^) under four UV-A lamps (Sankyo Denki, Japan) to remove residual photoreducible Hg­(II) and Hg(0) in the working solution. Subsequently, Hg–DOM equilibrated solution, prepared as described in Text S1 using the Hg-removed reaction matrix, was introduced into a quartz reactor used in previous studies,^4,6^ and purged with Hg-free air under dark conditions to remove any remaining Hg(0). Photoreduction experiments were then conducted for 2–37 h following calibration with an internal Hg permeation source. The outgoing air from the solution was passed through a soda-lime tube and a 0.2-μm PTFE filter assembly to remove water vapor and coarse particles. The Hg(0) released from the reaction solution was alternately collected on two gold cartridges and thermally desorbed at 550 °C using a Tekran 2537X analyzer. Purging continued until no Hg(0) was detected, indicating that the Hg(0) concentrations fell below the instrumental detection limit (<0.1 ng m^–3^). This procedure minimizes Hg(0) reoxidation,^6,25^ thereby allowing accurate determination of the gross *k*_r_. Potential loss of Hg(0) due to wall adsorption during purging is expected to be minimal, given the low affinity of quartz for Hg(0) adsorption.^3^ The relative percent difference of duplicate *k*_r_ measurements and the relative standard deviation of triplicate measurements were 21 ± 17% (*n* = 30) and 12 ± 8% (*n* = 3), respectively. The method detection limit for *k*_r_, determined as 3.365 × standard deviation of replicate *k*_r_ measurements (*n* = 6) in ASW containing 2 pM Hg­(II) and 40 nM GSH, was 0.054 h^–1^.

Previous studies have reported that *k*_r_ values for Hg­(II) photoreduction, representative of the upper few centimeters of the surface ocean, were approximately 0.25 h^–1^ under UV-A irradiation and remained invariant with UV intensity within the natural UV range of the ocean mixed layer.^6^ In contrast, *k*_r_ values measured under UV–B were higher at the surface (∼0.35 h^–1^) but decreased substantially with depth as UV intensity attenuated, indicating that UV-A is likely more effective than UV–B for driving Hg­(II) photoreduction throughout the mixed layer.^6^ Considering these findings, UV-A irradiation was employed in the present study. The intensity of UV-A irradiance incident on the solution was 14.6 W m^–2^, determined using a ferrioxalate actinometer (Figure S1; Text S2). This value is comparable to the monthly averaged UV-A irradiance within the upper 5 m water column (11–18 W m^–2^), estimated based on the incoming UV-A irradiance of 18 W m^–2^ at sea surface adjusted for atmospheric attenuation by ozone and cloud cover in the midlatitude (30°–40°N) in August. Vertical attenuation within the water column was then assessed using the diffuse attenuation coefficient at 340 nm (*K*_d340_ = 0.10 m^–1^), which was estimated using an empirical relationship of *K*_d380_ (0.061 m^–1^), derived from Moderate Resolution Imaging Spectroradiometer (MODIS-Aqua) observations collected from August in 2002 to 2025.^26^ The temperature inside the photoreactor was maintained at 25.5 ± 1.2 °C (*n* = 59) using an internal circulation fan.

Overall photoreduction experiments employed three matrices, Type-1 DIW, PBS, and ASW. A 1 mM PBS was prepared by diluting 0.1 M stock PBS containing 94 mM K_2_HPO_4_ and 6.5 mM KH_2_PO_4_. ASW was prepared by dissolving major inorganic sea salts in DIW without buffering, to achieve a salinity of 35 PSU (Table S1). Details on experimental variables for seawater matrix are included in the Supporting Information (Text S3). For each matrix, total alkalinity and dissolved inorganic carbon (DIC) concentration were measured using potentiometric titration and coulometric analysis, respectively,^27,28^ using a VINDTA system (Marianda, Kiel, Germany). Throughout the measurement period, analytical precision was approximately ±2 μmol kg^–1^ for both, based on comparisons with certified reference seawater standards (A. Dickson, Scripps Institution of Oceanography). The pH decreased by 1.4 to 7.5% after 12 h of aeration in DIW, PBS, and natural seawater due to dissolution of inorganic carbon, as confirmed by increased DIC levels (Table S2). Although DIC changes were moderate in DIW (35%) and natural seawater (13%), a substantial increase (800%) was observed in PBS due to the enhanced bicarbonate solubility. Nonetheless, obtained DIC values were much lower than seawater concentration. The pH and DIC showed opposite trends in ASW because bicarbonate was removed during aeration; however, overall changes remained within 6% of initial values. Dissolved oxygen (DO) concentrations increased from 9% to 52% after 12 h aeration (Table S2). Elevated DO concentration can affect *k*_*r*_ due to the production of ^1^O_2_ and O_2_^•–^ from ^3^DOM* and O_2_.^18,21^ In fact, enhanced Hg­(I) oxidation rate constants related to DO increase have been reported in the literature (e.g., 0.31 h^–1^ at 7.9 mg L^–1^ and 0.71 h^–1^ at 21 mg L^–1^ at pH 4.0).^29^ Although the Hg­(I) oxidation rate constant is unlikely to increase significantly as DO increases from 9% to 52%, it should be noted that *k*_*r*_ measured with aeration may be slightly underestimated compared to consistent DO conditions.

To evaluate the effect of thiols on *k*_r_, we employed cysteine (CYS) and glutathione (GSH) as representative biogenic aliphatic thiols commonly present in surface seawater. Reported concentrations range from 0.5–1.4 nM for CYS and 0.03–0.21 nM for GSH in the surface waters of the Pacific Ocean.^30^ We also included thioglycolic acid (TGA) as a nonbiogenic but photoreactive aliphatic thiol, as it can be produced by anaerobic degradation of DOM.^12^ 2-mercaptophenol (2-MP), thiosalicylic acid (TSA), and 4-mercaptobenzoic acid (4-MBA) were included to represent photoreactive aromatic thiol compounds (Figure S2; Text S1). In addition, anthranilic acid, 4-aminobenzoic acid, salicylic acid, 4-nitrophenol, 2-nitrophenol, and *p*-benzoquinone were selected as representative aromatic DOM analogues, derived from terrestrial DOM and plankton metabolism, that are not expected to complex with Hg­(II) under typical seawater concentrations of chloride (∼0.54 M) and thiols (<10 nM) (Figure S2; Text S4).^20,31^ Detailed descriptions of the experimental variables for thiolic and nonthiolic DOMs, as well as seawater matrix preparation, are provided in the Supporting Information (Table S1; Texts S1 and S4).

### 2.2 Kinetic Modeling for Hg­(II) Photoreduction

The *k*_r_ values of reducible Hg­(II) complexed with inorganic ligands (e.g., OH^–^ and Cl^–^) or thiols were estimated using pseudo-first order kinetics as follows.^6^

Hg(II)+hv→Hg(0)

$$\frac{d{\left[\mathrm{Hg}\left(\mathrm{II}\right)\right]}_{t}}{dt}=-k_{r}{\left[\mathrm{Hg}\left(\mathrm{II}\right)\right]}_{t}$$

$$\ln\frac{{\mathrm{Hg}\left(\mathrm{II}\right)}_{t}}{{\mathrm{Hg}\left(\mathrm{II}\right)}_{0}}=-k_{r}t$$

$${\mathrm{Hg}\left(\mathrm{II}\right)}_{t}={\mathrm{Hg}\left(\mathrm{II}\right)}_{0}-{\mathrm{Hg}\left(0\right)}_{t}={\mathrm{Hg}\left(\mathrm{II}\right)}_{0}\times e^{{-k}_{r}t}$$

$${\mathrm{Hg}\left(0\right)}_{t}={\mathrm{Hg}\left(\mathrm{II}\right)}_{0}\times \left(1-e^{{-k}_{r}t}\right)$$

Here, Hg­(II)*_t_* and Hg­(II)_0_ represent the reducible Hg­(II) in solution at times *t* and 0, respectively, while Hg(0)*_t_* is the photoproduced Hg(0) at time *t*. The *k*_r_ was predicted by a curve fitting using Sigma Plot 12.0 software.

## 3 Results and Discussion

### 3.1 Photoreduction of Hg­(II)-Thiols in DIW and PBS

The *k*_r_ values and reducible Hg­(II) concentrations were measured under UV-A irradiation in the presence of 4 nM and 40 nM thiolic DOM in DIW and PBS (Figure 1a,b and Table S3). No detectable *k*_r_ of Hg­(II) was observed when aliphatic thiols (CYS, GSH, and TGA) were added to DIW or PBS at either concentration. The negligible *k*_r_ values of Hg­(II) complexed with aliphatic thiols are consistent with previously reported ranges for CYS (0.01–0.06 h^–1^) and alkanethiols (0.00030–0.00072 h^–1^) in DIW.^13,23^ The slow photoreduction observed in the presence of aliphatic thiols can be attributed to their low UV-A absorption efficiencies (Figure 2) and the very low quantum yields (<10^–6^) of Hg­(II)–aliphatic thiol complexes.^13^

**Figure 1:**
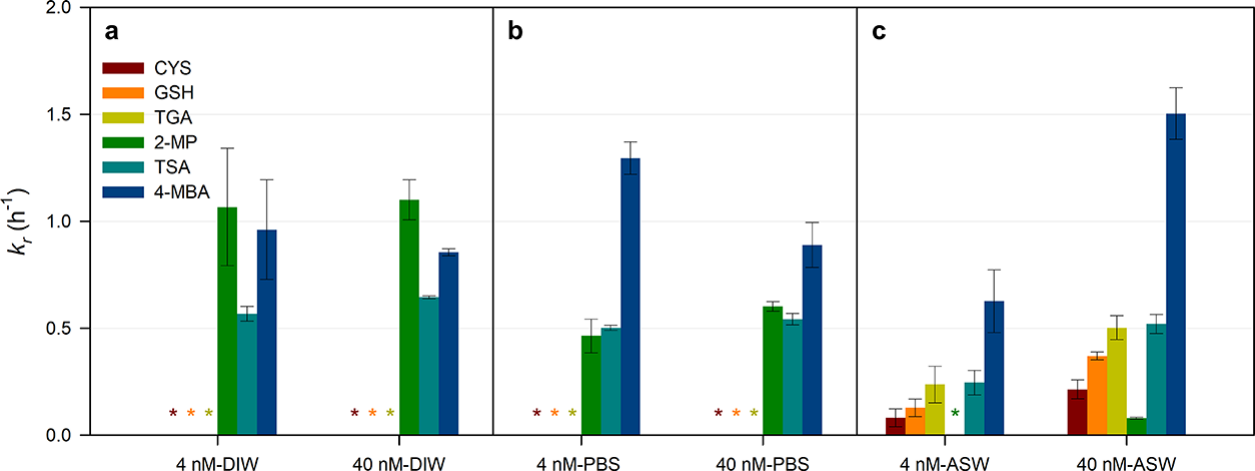
*k*_r_ of Hg­(II) in (a) DI water (DIW), (b) phosphate buffer solution (PBS), and (c) artificial seawater (ASW) with three aliphatic thiol ligands, cysteine (CYS), glutathione (GSH), and thioglycolic acid (TGA); and three aromatic thiol ligands, 2-mercaptophenol (2-MP), thiosalicylic acid (TSA), and 4-mercaptobenzoic acid (4-MBA) under UV-A irradiation. Asterisk (*) denotes that *k*_r_ values are under the detection limit. Error bar represents a standard deviation of *k*_r_.

**Figure 2:**
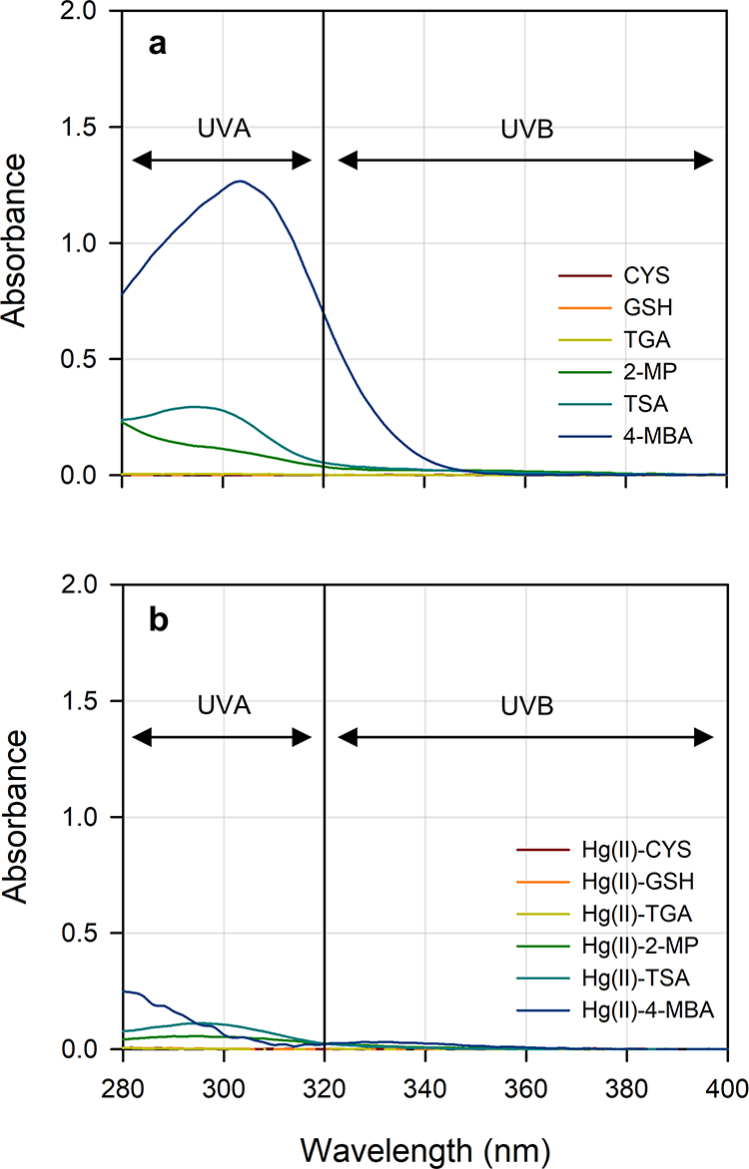
UV absorption spectra of (a) 0.08 mM free thiols and (b) 0.01 mM Hg­(II)–thiol complexes in 5 mM phosphate buffer solution. The UV absorption spectra of Hg­(II)–thiol complexes in (b) are derived by subtracting the UV absorbance of 0.08 mM thiol from that of the experimental solutions containing 0.1 mM thiol and 0.01 mM HgCl_2_.

The *k*_r_ values of Hg­(II) ranged from 0.57 to 1.1 h^–1^ when 4 nM aromatic thiols (2-MP, TSA, and 4-MBA) were added to DIW—substantially higher than those obtained with aliphatic thiols (Figure 1a). A comparable range (0.46–1.3 h^–1^) was observed in PBS (Figure 1b). The markedly higher *k*_r_ values for aromatic thiols suggest that photoreduction of Hg­(II)–aromatic thiol complexes may proceed predominantly via direct photolysis, which is consistent with their strong UV-A absorptivity (Figure 2). This interpretation is further supported by the minimal dependence of *k*_r_ on aromatic thiol concentration (Figure 1a,b). When the concentrations of aromatic thiols increased from 4 to 40 nM, *k*_r_ values for 2-MP, TSA, and 4-MBA in DIW remained comparable. A similar trend was observed for 2-MP and TSA in PBS, whereas 4-MBA exhibited a significant decrease in *k*_r_ at 40 nM. These results are consistent with previous findings that direct photolysis of Hg­(II) and other organic pollutants exhibits little to no concentration dependence when active photosensitizers such as humic substances and reactive oxygen species, including ^1^O_2_, are present.^12,32^

### 3.2 Photoreduction of Hg­(II)-Thiols in ASW

In ASW, the *k*_r_ of Hg­(II) increased substantially compared to DIW and PBS in the presence of aliphatic thiols (Figure 1c). For 4 nM thiol amendments, the *k*_r_ values were 0.081 h^–1^ for Hg–CYS, 0.13 h^–1^ for Hg–GSH, and 0.24 h^–1^ for Hg–TGA. This trend in *k*_r_ corresponds to the order of the thiol p*K*_a_ values: CYS (8.4) < GSH (9.2) < TGA (10.6).^33,34^ A higher p*K*_a_ indicates greater nucleophilicity of RS^–^,^35^ which facilitates stable nucleophilic attack on electrophilic species such as ^•^OH and CO_3_^•–^ to generate RS^•^ (eq 1).^36^ In addition, the GSH thiyl radical exhibits a lower standard redox potential [*E* ^o^ (RS^•^, H^+^/RSH) = 1.16 V] than that of CYS [*E* ^o^ (RS^•^, H^+^/RSH) = 1.20–1.39 V],^37^ suggesting that redox thermodynamics favor GSH oxidation over CYS oxidation when coupled to Hg­(II) reduction.

1 $${\mathrm{RS}}^{-}+\mathrm{electrophile}\to {\mathrm{RS}}^{•}$$

2 $${\mathrm{RS}}^{•}+{\mathrm{RS}}^{-}\to {\mathrm{RSSR}}^{•-}$$

3 $${\mathrm{RRSR}}^{•-}+O_{2}\to {O_{2}}^{•-}$$

Once RS^•^ is generated, it reacts with RS^–^ to form disulfide radical anions (RSSR^•–^, eq 2), which act as potential reductants with standard redox potentials, *E* ^o^ (RSSR/RSSR^•–^), ranging from −2.06 to −1.12 V.^38^ RSSR^•–^ can subsequently reduce O_2_ to O_2_^•–^, with *E* ^o^ (O_2_/O_2_^•–^) = −0.18 V (eq 3).^39^ In this radical system, the steady-state concentrations of the one-electron reductants, O_2_^•–^ and RSSR^•–^, are expected to increase in the order of p*K*_a_ values (CYS < GSH < TGA), which accounts for the observed trend in *k*_r_. Furthermore, *k*_r_ of Hg­(II) increased when aliphatic thiol concentrations in ASW were raised from 4 to 40 nM, reflecting enhanced RS^–^ availability (Figure 1c). The photoreduction of Hg­(II) complexed with aliphatic thiols in ASW appears to proceed primarily via indirect photolysis,^14–16^ being positively influenced by availability and nucleophilicity of RS^–^.

In ASW, the *k*_r_ of Hg­(II) in the presence of 4 nM aromatic thiols (2-MP, TSA, and 4-MBA) was below the detection limit for 2-MP, 0.25 h^–1^ for TSA, and 0.63 h^–1^ for 4-MBA (Figure 1c), all lower than the corresponding values measured in DIW and PBS. The reduction in *k*_r_ in ASW is likely associated with the formation of HgCl_*x*_ species, which exhibit substantially lower *k*_r_ than Hg­(SR) and Hg­(SR)_2_.^10,11^ Thermodynamic calculations indicate that a small fraction of total Hg existed as HgCl_*x*_ in the presence of 4 nM TSA, explaining the lower *k*_r_ in ASW compared to DIW and PBS (Table S4). In the same context, when TSA concentration increased to 40 nM, the HgCl_*x*_ fraction decreased to nearly zero, and *k*_r_ became comparable to that in DIW and PBS.

The observed order of *k*_r_ (2-MP < TSA < 4-MBA) aligns with the UV-A absorptivity of the thiols (Figure 2), confirming that photoreduction proceeds predominantly via direct photolysis, as in DIW and PBS. At 40 nM, *k*_r_ in ASW was 0.08 h^–1^ for 2-MP, 0.52 h^–1^ for TSA, and 1.50 h^–1^ for 4-MBA, following the UV absorptivity trend. Notably, *k*_r_ for 2-MP increased from below the detection limit at 4 nM to 0.08 h^–1^ at 40 nM, and similar increases were observed for TSA and 4-MBA. This enhancement could be attributed to reduced formation of HgCl_*x*_ at higher thiol concentrations, consistent with LMCT-driven photoreduction. A similar effect has been reported for MeHg, where higher thiol concentrations in coastal seawater promoted direct photolysis by increasing the fraction of MeHg-thiol species.^40^

### 3.3 Role of Sea Salts: Chloride and Bicarbonate

To investigate the effect of sea salts on Hg­(II) photoreduction, *k*_r_ of inorganic Hg­(II) was measured in three different matrices: 0.4 M NaCl, a mixed solution of 0.4 M NaCl and 2 mM NaHCO_3_, and ASW. In all cases, *k*_r_ was below the detection limit (Figure 3a and Table S5). Thermodynamic calculations indicate that Hg­(II) was predominantly present as Hg–Cl complexes (>99%) in these solutions (Table S6), confirming that photoreduction is strongly suppressed when Hg­(II) is primarily complexed as chloride in the absence of thiolic DOM.^11^ This suppression likely results from two factors: (i) HgCl_4_^2–^ absorbs UV light mainly in the UV–C region (<280 nm), which is absent in the present study, and (ii) rapid reoxidation of Hg­(I) to Hg­(II) by chlorine radicals (Cl^•^) and dichloride radical anions (Cl_2_^•–^), as described in eqs 4–7.^11,41^

4 HgCl42−+hν→H•gCl32−+Cl•

5 $${\mathrm{Cl}}^{•}+{\mathrm{Cl}}^{-}\to {{\mathrm{Cl}}_{2}}^{•-}$$

6 H•gCl32−+Cl•→HgCl42−

7 H•gCl32−+Cl2•−→HgCl42−+Cl−

To simulate Hg­(II) photoreduction under natural seawater conditions, 40 nM GSH was added as a model DOM ligand, since Hg­(II) in the surface ocean is expected to bind to biogenic low-molecular-weight thiols such as GSH and CYS.^42^ The *k*_r_ of Hg–GSH was then measured in the presence of individual sea salt components at seawater concentrations in PBS (Figure 3c). Interestingly, *k*_r_ was below the detection limit for all salts except 2 mM NaHCO_3_, indicating that bicarbonate enhances the photoreduction of Hg­(II)–GSH. To verify this effect, *k*_r_ was measured in three water matrices: 0.4 M NaCl, a mixed solution of 0.4 M NaCl and 2 mM NaHCO_3_, and ASW (Figure 3b). As a result, *k*_r_ was negligible in the NaCl solution, likely due to the reoxidation of Hg­(I) by Cl^•^ and Cl_2_^•–^ radicals as described below.

8 Hg(SR)2+hν→H•g(SR)+RS•

9 H•g(SR)+Cl•−→Hg(SR)Cl

10 H•g(SR)+Cl2•−→Hg(SR)Cl+Cl−

The *k*_r_ increased to 0.37 h^–1^ in the mixed solution of NaCl and NaHCO_3_ and to a similar value (0.37 h^–1^) in ASW. We suggest that this increase is likely because HCO_3_^–^ and CO_3_^2–^ scavenge Cl^•^ and Cl_2_^•–^ and produce HCO_3_^•^ and CO_3_^•–^, thereby enhancing *k*_r_ of Hg-GSH, as described in eqs 11–16.^43^

11 $${{\mathrm{HCO}}_{3}}^{-}/{{\mathrm{CO}}_{3}}^{2-}+{\mathrm{Cl}}^{•}/{{\mathrm{Cl}}_{2}}^{•-}\to {\mathrm{HCO}}_{3}^{•}/{{\mathrm{CO}}_{3}}^{•-}$$

12 $${\mathrm{RS}}^{-}+{\mathrm{HCO}}_{3}^{•}/{{\mathrm{CO}}_{3}}^{•-}\to {\mathrm{RS}}^{•}+{{\mathrm{HCO}}_{3}}^{-}/{{\mathrm{CO}}_{3}}^{2-}$$

13 $${\mathrm{RS}}^{•}+{\mathrm{RS}}^{-}\to {\mathrm{RSSR}}^{•-}$$

14 $${\mathrm{RSSR}}^{•-}+O_{2}\to {O_{2}}^{•-}$$

15 $$\mathrm{Hg}\left(\mathrm{II}\right)+{\mathrm{RSSR}}^{•-}/{O_{2}}^{•-}\to \mathrm{Hg}\left(I\right)+\mathrm{RSSR}/O_{2}$$

16 $$\mathrm{Hg}\left(I\right)+{\mathrm{RSSR}}^{•-}/{O_{2}}^{•-}\to \mathrm{Hg}\left(0\right)+\mathrm{RSSR}/O_{2}$$

We found that the upper-limit rate constants for reactions of Cl_2_^•–^ and Br_2_^•–^ with phenolic DOM are 10^1^–10^3^ L (mg C)^−1^ s^–1^. In comparison, the rate constants for reactions of Cl_2_^•–^ and Cl^•^ with CO_3_^2–^/HCO_3_^–^ are ∼10^8^ M^–1^ s^–1^.^44^ Hence, the DOM concentration would need to exceed 10^2^ mg L^–1^ to act as a dominant sink for Cl_2_^•–^ and Cl^•^ at a bicarbonate concentration of 2 mM. However, DOM concentrations in natural seawater typically range from 1.2 to 2.4 mg L^–1^, indicating that bicarbonate, rather than DOM, likely plays a major role in halogen radical scavenging. Nonetheless, direct measurements of steady-state concentrations of carbonate and chlorine radicals in seawater are needed to support this interpretation.

**Figure 3:**
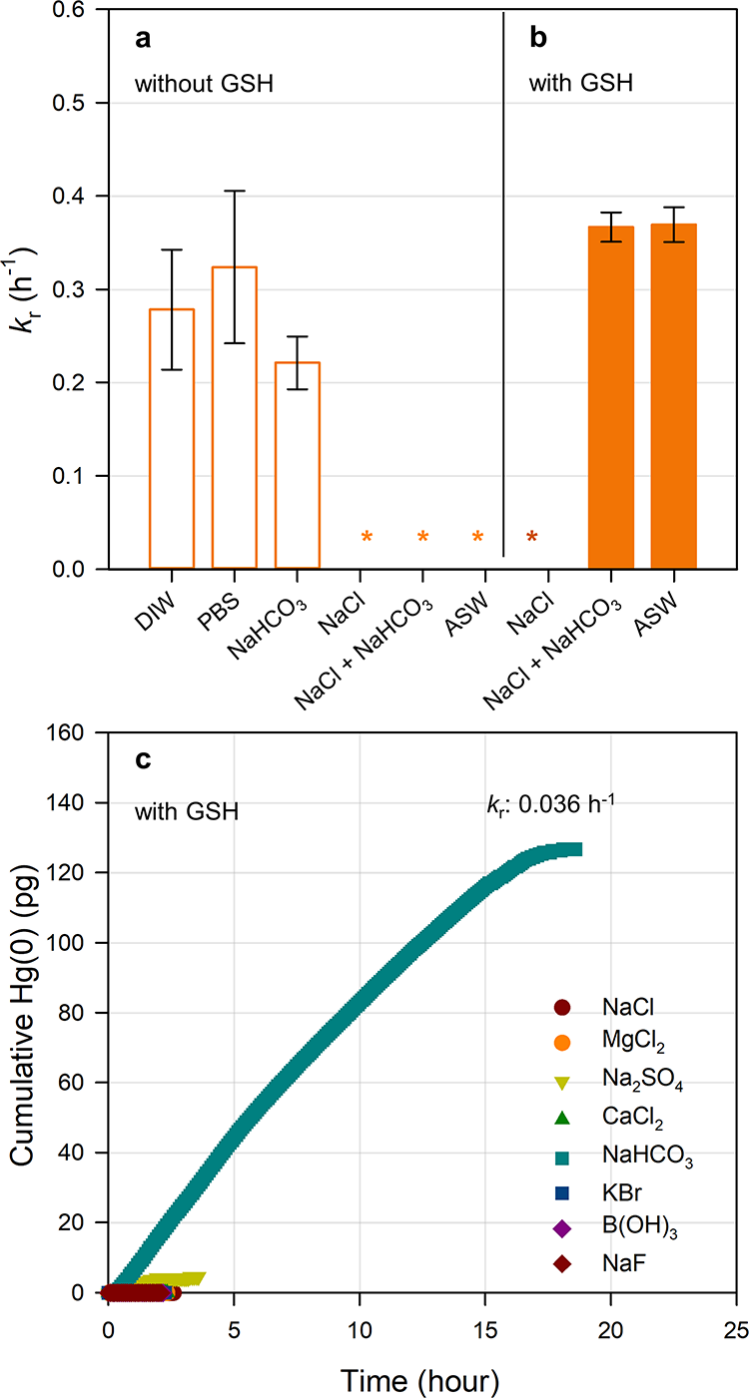
(a) The *k*_r_ of inorganic Hg­(II) in DI water (DIW), 1.0 mM phosphate buffer solution (PBS), 2.0 mM NaHCO_3_ solution. 0.4 M NaCl solution, a mixed solution of 0.41 M NaCl and 2.0 mM NaHCO_3_, and artificial seawater (ASW) under UV-A. (b) The *k*_r_ of Hg­(II) in 0.41 M NaCl solution, a mixed solution of 0.41 M NaCl and 2 mM NaHCO_3_, and ASW in the presence of GSH under UV-A. (c) Cumulative Hg(0) production obtained using Hg­(II), GSH, and sea salt component dissolved in 1 mM PBS at pH 8 under UV-A. Asterisk (*) denotes under the detection limit.

The pivotal role of bicarbonate in enhancing, and chloride in suppressing, *k*_r_ of Hg­(II) in ASW in the presence of 40 nM GSH was confirmed by examining the effect of varying bicarbonate and chloride concentrations (Figure S3). Excessive production of oxidants mediated by chloride is evidenced by the extended delay in Hg(0) formation in 0.7 M NaCl solution (Figure S3b). The *k*_r_ of Hg–GSH in ASW increased with HCO_3_^–^ concentration, from 1.8 × 10^–7^ h^–1^ at 0.2 mM to 0.038 h^–1^ at 1 mM, and 0.37 h^–1^ at 2 mM (Figure S3a). At low bicarbonate (0.2 mM), Hg(0) formation was delayed due to reoxidation of Hg­(I) by Cl^•^ and Cl_2_^•–^.^7,11^ In contrast, at 2 mM NaHCO_3_, chloride-mediated oxidation was effectively suppressed, likely via bicarbonate-facilitated formation of RS^•^ and subsequent reduction of Hg­(II) (eqs 11–16), allowing Hg(0) to be produced in ASW. Notably, this initial lag phase has not been observed in the photoreduction of Hg­(II) in natural seawater (Figure S3c) or in previous studies.^4,6^ Considering that DIC in natural seawater typically ranges from 1.8 to 2.3 mM,^45^ the rapid onset of Hg(0) photoproduction is likely driven by the combined effects of carbonate and chloride. The steady-state concentrations of Cl^•^ and Cl_2_^•–^ appear to substantially reduced by reactions with HCO_3_^–^/CO_3_^2–^ and DOM,^44^ thereby suppressing halogen radical-mediated oxidation of Hg­(I) in natural seawater.

### 3.4 Role of Nonbinding Aromatic DOM

The effects of nonbinding aromatic DOM on the photoreduction of Hg­(II)–thiols were evaluated in ASW (Figure 4 and Table S7), as aromatic DOM—commonly found in humic and fulvic acids—has been strongly linked to variations in *k*_r_,^46^ and can generate both oxidizing intermediates (^•^OH, ^1^O_2_, and ^3^DOM*)^18^ and reducing intermediates (O_2_^•–^ and organic radicals).^20,47^ CYS was used as a model thiolic ligand, and thermodynamic calculations indicated that Hg­(CYS)­H^+^ (54%) and Hg­(CYS) (30%) were the dominant Hg­(II) species in the presence of 40 nM CYS in ASW (Table S4). Among three tested benzoic acid derivatives, *k*_r_ decreased in the order anthranilic acid (0.24 h^–1^) > 4-aminobenzoic acid (0.18 h^–1^) > salicylic acid (0.13 h^–1^) (Figure 4b). Similar trends, but with higher *k*_r_ values (0.6–8.4 h^–1^), were reported for the reduction of HgCl_2_ by these molecules.^20^ The higher literature values may reflect stronger UV-A intensity and lower O_2_ concentrations, which enhance steady-state concentrations of organic radicals.^20^ In the present study, 4-aminobenzoic acid and salicylic acid exhibited lower *k*_r_ than the control (without aromatic DOM), suggesting that reactive intermediates (e.g., ^•^OH, ^1^O_2_, and ^3^DOM*) reoxidized Hg(0) or Hg­(I) to Hg­(II), thereby suppressing photoreduction. Conversely, anthranilic acid showed *k*_r_ comparable to the control, indicating that produced oxidants were mostly scavenged,^48^ or that the negative effects of oxidants formation were offset by the positive effects of reductants formation (e.g., O_2_^•–^ and organic radicals).^20^

**Figure 4:**
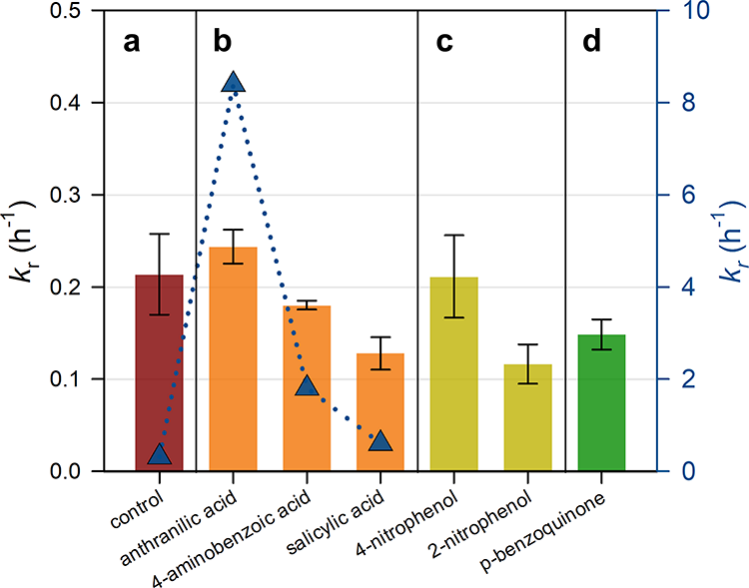
*k*_r_ values in ASW for reduction of Hg–cysteine (CYS) (a) without aromatic DOM; (b) with benzoic acid-type molecules, (c) nitrophenol-type molecules, and (d) p-benzoquinone. Hg­(II) and CYS concentrations were 2 pM and 40 nM, respectively. Error bar represents a standard deviation of measured *k*_r_ values available in Table S7. *k*_r_ for reduction of HgCl_2_ with benzoic-acid type molecules are shown with a blue dotted line.

The effect of nitrophenols on the photoreduction of Hg­(CYS) was evaluated as another representative nonbinding, photoreactive aromatic DOM (Figure 4c). The *k*_r_ of Hg-CYS in the presence of 4-nitrophenol was comparable to the control, whereas 2-nitrophenol decreased *k*_r_ by 46%. 4-Nitrophenol exhibits higher UV-A absorptivity and photochemical quantum yield—2 orders of magnitude greater than 2-nitrophenol under UV/vis irradiation (280–500 nm)—suggesting more efficient initiation of Hg­(II) photoreduction through enhanced production of O_2_^•–^ and organic radicals.^49^

Based on the results described in the previous sections, we hypothesize that reactive oxidants, such as ^•^OH, ^1^O_2_, and ^3^DOM*,^18^ produced via photoactivation of aromatic DOM, can reoxidize Hg(0) or Hg­(I) to Hg­(II), thereby decreasing *k*_r_ of Hg–CYS. Because Hg(0) was continuously purged during UV-A irradiation, reoxidation of Hg(0) is unlikely; thus, oxidation of Hg­(I) could be the primary pathway for the observed decrease in *k*_r_ in the presence of aromatic DOM. To test if oxidant intermediates are produced from aromatic DOM, scavengers of reactive oxygen and DOM species—2-propanol for ^•^OH, furfuryl alcohol for ^1^O_2_, and sorbic acid for ^3^DOM*—were added to Hg–CYS solutions in the absence and presence of 4-aminobenzoic acid and salicylic acid (Figure 5a and Table S7). *k*_r_ increased upon scavenger addition, confirming that Hg­(I) reoxidation drives the observed decreases in *k*_r_. The increases were smaller in the control (51% with 2-propanol, 116% with furfuryl alcohol, and 110% with sorbic acid) compared to salicylic acid (264%, 293%, and 266%, respectively) and 4-aminobenzoic acid (113%, 166%, and 188%, respectively) treatments, indicating that aromatic DOM enhances photogeneration of intermediate oxidants. The effect was more pronounced for furfuryl alcohol and sorbic acid than 2-propanol, and this suggests that ^1^O_2_ and ^3^DOM* are the dominant oxidants responsible for Hg­(I) photooxidation in Hg–CYS complexes. The similar increases observed for furfuryl alcohol and sorbic acid are consistent with the fact that ^3^DOM* is a primary source of ^1^O_2_ in natural water.^18^ The addition of all three scavengers produced the largest increases in *k*_r_ for Hg–CYS, demonstrating that Hg­(I) photooxidation relies on multiple reactive oxidants.

**Figure 5:**
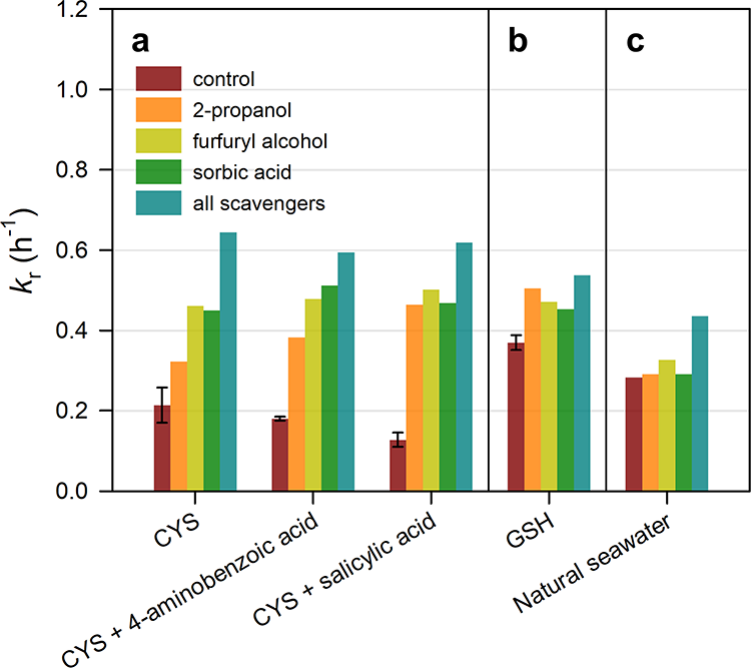
(a) The *k*_r_ for Hg–cysteine (CYS) with various nonbinding aromatic DOMs and oxidant scavengers in artificial seawater; (b) for Hg–glutathione (GSH) with oxidant scavengers in artificial seawater; and (c) for Hg­(II) in natural seawater with oxidant scavengers. Error bar represents a standard deviation of measured *k*_r_ values.

The scavenger addition had a smaller effect on *k*_r_ for Hg–GSH than for Hg–CYS (Figure 5a,b), likely due to greater oxidant production (e.g., ^1^O_2_) from oxygen-dependent chain reactions with CYS and the higher fraction of electron-rich RS^–^ in CYS compared to GSH.^33,36^ The addition of all three scavengers also resulted in the largest increases in *k*_r_ for the Hg–GSH system compared with a single scavenger. Notably, scavenger addition had negligible effects on *k*_r_ in natural seawater relative to Hg–CYS and Hg–GSH systems for the single scavenger case (Figure 5c), indicating that Hg­(I) reoxidation is suppressed by natural DOM, where phenolic and sulfur-containing moieties can efficiently scavenge ^3^DOM* and ^1^O_2_.^18,50^

### 3.5 Photoreduction Pathways of Hg­(II) in Seawater

A two-step, one-electron transfer mechanism [Hg­(II) → Hg­(I) → Hg(0)] has been proposed as a plausible pathway for Hg­(II) photoreduction in aqueous solution, considering the potential contribution of one-electron reductants (e.g., RSSR^•–^ and O_2_^•–^) and the energetic limitation of direct two-electron transfer.^14,24^ Furthermore, the results of scavenger experiments indicate that Hg­(I) reoxidation to Hg­(II) must be incorporated into kinetic descriptions of Hg­(II) photoreduction, particularly in the presence of aliphatic thiols and aromatic DOM. Accordingly, we extended the single-step irreversible kinetic model (*k*_r_) to a two-step model consisting of consecutive one-electron reduction steps (*k*_1_ and *k*_2_), with a reversible first step (*k*_–1_), to explicitly account for Hg­(I) reoxidation (eq 17).

17 $$\mathrm{Hg}\left(\mathrm{II}\right)\overset{k_{1}}{\underset{k_{-1}}{↔}}\mathrm{Hg}\left(I\right)\overset{k_{2}}{\to }\mathrm{Hg}\left(0\right)$$

The analytical solution for Hg(0) as a function of time and the associated rate coefficients are described in Text S5. Initially, *k*_1_ and *k*_2_ were estimated by fitting the model to the Hg(0)–time curves obtained from reactions conducted in the presence of three scavengers (i.e., ^•^OH, ^1^O_2_, and ^3^DOM*) (eq 18).

18 $$\mathrm{Hg}\left(\mathrm{II}\right)\underset{k_{1}}{\to }\mathrm{Hg}\left(I\right)\underset{k_{2}}{\to }\mathrm{Hg}\left(0\right)$$

This approach isolates the forward reduction from the reverse oxidation step by assuming that intermediate oxidants are completely removed by three scavengers, thereby minimizing Hg­(I) reoxdation.^51^ The analytical solution for eq 18 is provided in Text S5. We assigned a lower value to *k*_1_ and a higher value to *k*_2_, while switching *k*_1_ and *k*_2_ yielded the same goodness of fit of the model (*R* ^2^) (Table 1). The standard redox potentials (*E* ^o^) for the Hg­(II)/Hg­(I) and Hg­(I)/Hg(0) couples are 0.91 and 0.80 V, respectively.^52,53^ Therefore, reduction of Hg­(II) to Hg­(I) is thermodynamically more favorable than reduction of Hg­(I) to Hg(0). However, the first electron-transfer step is known to involve a high activation energy barrier associated with solvation energy changes and structural rearrangements, in addition to the inherent instability of Hg­(I).^44^ Moreover, the redox potential of the Hg­(II)/Hg­(I) couple is expected to decrease when Hg­(II) is complexed with chloride and thiols in seawater.^29^

**Table 1:** Estimated Rate Constants For The Reduction of Hg­(II) to Hg­(I) (*k*_1_) and Hg­(I) to Hg(0) (*k*_2_), and the Oxidation of Hg­(I) to Hg­(II) (*k*_–1_) Based on the Two-Step Reversible Kinetic Model, and *k*_r_ Based on the One-Step Irreversible Kinetic Model^a^

			two-step reversible model				one-step irreversible model
ligand and medium	DOM	scavenger	*k*_1_ (h^–1^)	*k*_2_ (h^–1^)	*k*_–1_ (h^–1^)	*R* ^2^	*k*_r_ (h^–1^)
CYS in ASW	none	P + F + S	0.88	4.4		1.0	0.65
		none	0.88	4.4	14	0.98	0.16
		none	0.88	4.4	7.6	0.97	0.25
		none	0.88	4.4	12	0.96	0.23
	4-aminobenzoic acid	P + F + S	0.87	3.2		0.99	0.59
		none	0.87	3.2	8.3	0.99	0.18
		none	0.87	3.2	12	0.88	0.18
	salicylic acid	P + F + S	0.85	3.6		0.99	0.62
		none	0.85	3.6	15	0.98	0.12
		none	0.85	3.6	11	0.96	0.14
GSH in ASW	none	P + F + S	0.60	13		1.0	0.54
		none	0.60	13	6.3	0.99	0.35
		none	0.60	13	4.9	0.99	0.37
		none	0.60	13	7.4	0.99	0.39
natural seawater	none	P + F + S	0.58	9.9		0.99	0.44
		none	0.58	9.9	7.7	0.99	0.28

a The *R* ^2^ denotes goodness of fit of the model. P + F + S: 2-propanol, furfuryl alcohol, and sorbic acid.

Once *k*_1_ and *k*_2_ were determined, *k*_–1_ was estimated from control reactions without scavengers, assuming they do not alter the fundamental forward reduction pathways (Table 1). This assumption holds if the scavengers do not affect RSSR^•–^ and O_2_^•–^ in eqs 15, 16. Since ^3^DOM* is quenched by sorbic acid via energy transfer, it is not expected to affect RSSR^•–^ and O_2_^•–^ concentrations. However, reactions of 2-propanol with ^•^OH and of furfuryl alcohol with ^1^O_2_ can produce H_2_O_2_, which may react with residual ^•^OH to form O_2_^•–^.^54,55^ Nevertheless, the residual ^•^OH concentrations would be extremely low under excessive addition of 2-propanol. If the addition of the three scavengers is insufficient to completely inhibit the Hg­(I) oxidation, then the true *k*_1_ and *k*_–1_ values may be higher and the true *k*_2_ value lower than those reported in Table 1. Accordingly, *k*_–1_ and *k*_1_ values in Table 1 should be regarded as lower bounds, and *k*_2_ as an upper bound, of the uncertainty range of the true values. Based on this limitation, direct measurements of the rate constants, which require lowering the detection limit for Hg­(I) from the current range of (0.05–1.1 nM)^29,56^ to the subpicomolar level, are needed for precise understanding of the redox kinetic mechanisms.

For Hg–CYS and Hg–GSH in ASW, *k*_1_ ranged from 0.60 to 0.88 h^–1^, *k*_2_ from 3.2 to 13 h^–1^, and *k*_–1_ from 4.9 to 15 h^–1^ (Table 1). In natural seawater, *k*_1_, *k*_2_, and *k*_–1_ were 0.58, 9.9, and 7.7 h^–1^, respectively, indicating that reduction of Hg­(II) to Hg­(I) is the rate-determining step and the rates of Hg­(I) reoxidation and reduction are comparable in ASW and natural seawater. We performed a sensitivity analysis for *k*_–1_ due to the large variability observed in replicate tests conducted without scavengers (Table S8). The results show that *k*_–1_ changes by 13–18% for a ±10% change in *k*_1_ and by 10–15% for a ±10% change in *k*_2_, suggesting that accurate measurements of *k*_1_ and *k*_2_ are necessary for reliable estimation of *k*_–1_.

The *k*_1_ values were comparable between CYS and GSH in ASW, whereas *k*_–1_ values were consistently higher for CYS than for GSH, suggesting that Hg­(I) is more efficiently reoxidized to Hg­(II) in the presence of CYS than GSH, likely due to enhanced ^1^O_2_ production via the self-condensation of peroxysulphenyl radicals (RSOO^•^), which are generated through the reaction of O_2_ with cysteinyl radicals (8.1 × 10^9^ M^–1^ s^–1^) at a rate faster than the reaction of O_2_ with glutathionyl radicals (1.6 × 10^9^ M^–1^ s^–1^).^33,36^ With and without DOM, *k*_1_ for Hg–CYS in ASW (0.88 h^–1^) was 3.5–7.3 times higher than the observed *k*_r_ (0.12–0.25 h^–1^), whereas for Hg­(II) in natural seawater, *k*_1_ (0.58 h^–1^) was only 2.1 times higher than *k*_r_ (0.28 h^–1^). This result reflects the weak Hg­(I) reoxidation to Hg­(II) in natural seawater, likely due to the scavenging of reactive intermediates by natural DOM.^18,50^ Overall, the two-step reversible model, which includes Hg­(I) as an intermediate, captures the redox dynamics among Hg­(II), Hg­(I), and Hg(0) in seawater. This model aligns with previous recommendations that Hg redox parametrizations should move beyond simplified one-step reversible reactions.^6^

## 4 Environmental Implications

Our findings suggest that Hg­(II) photoreduction kinetics are primarily controlled by thiolic and aromatic DOM, salinity, and DIC. Given the limited abundance of aromatic thiol moieties in DOM,^57^ aliphatic thiols (e.g., GSH and CYS) are expected to be the principal drivers of Hg­(II) photoreduction in seawater. This is consistent with the notably higher *k*_r_ values observed for Hg­(II) complexed with aromatic thiols in DIW and PBS (0.57–1.3 h^–1^), which largely exceed those reported for estuarine waters at salinities of 13.5–26.8 g L^–1^ (0.22–0.31 h^–1^) under UV-A irradiance (∼25 W m^–2^).^4^ The role of Hg–sulfide complexes in Hg­(II) photoreduction is likely negligible, as they exhibit low photochemical reactivity and tend to aggregate into nanoparticles exported to the deep ocean.^58^ Moreover, sulfide species rarely bind to Hg­(II) via photochemical pathways in surface seawater.^31^

In current global Hg models, the photoreduction rate of dissolved Hg­(II) in surface seawater is not described mechanistically in terms of seawater properties. Instead, these models apply the pseudo-first-order rate constant parametrized solely by light intensity.^9,59^ Our findings suggest that accurate prediction of Hg­(II) photoreduction rates requires parametrization of *k*_r_ as a function of thiolic and aromatic DOM concentrations, given their spatial variability between productive coastal waters and oligotrophic ocean waters. For example, thiol concentrations in coastal seawater are estimated at 17–26 μM, approximately four times higher than those in offshore waters (2.9–7.6 μM), based on observed DOC concentrations^3^ and reported RSH/DOC molar ratios from estuaries and continental shelves in North Atlantic (91 μmol mol^–1^)^60^ and Yellow Sea (32 μmol mol^–1^).^40^ Under these conditions, coastal waters should exhibit higher *k*_r_ values than offshore waters, reflecting the greater availability of RSSR^•–^ and O_2_^•–^. Consistently, *k*_r_ for Hg­(II) with 40 nM CYS was three times higher than with 4 nM CYS in ASW (Figure 1). Conversely, the photoreduction rate of Hg­(II) may decrease in coastal seawater, as tannin-like aromatic DOM from terrestrial vegetation can enhance *k*_–1_ and thereby reduce *k*_r_, due to its high electron-accepting capacity.^61^ However, *k*_–1_ is generally much larger than *k*_1_, and thus, the influence of aromatic DOM may be less critical than thiols in controlling Hg­(II) photoreduction rate in coastal waters. In fact, *k*_r_ values in coastal seawater (0.37 ± 0.062 h^–1^, *n* = 5) were approximately 1.3 times higher than those in offshore waters (0.29 ± 0.035 h^–1^, *n* = 5) in the marginal sea of the North Pacific,^3^ implying that the overall photoreduction rate of Hg­(II) is primarily determined by *k*_1_ rather than *k*_–1_.

The result of this study shows that salinity and DIC are significant parameters modulating Hg­(II) photoreduction rates. Therefore, parametrization of *k*_r_ using DIC and salinity, as well as aliphatic thiol and aromatic DOM concentrations, is necessary for accurate prediction of Hg­(II) photoreduction. This effect likely arises because DIC scavenges Cl^•^ and Cl_2_^•–^ and produces HCO_3_^•^ and CO_3_^•–^, which subsequently generate RS^•^. Elevated DIC concentrations are typically observed in high-latitude seawaters due to the enhanced CO_2_ solubility at low temperatures,^62^ and in upwelling regions, where vertical transport of deep water increases DIC concentrations in surface seawater.^45^ Ocean acidification has increased surface DIC levels by ∼1 μmol kg^–1^ relative to preindustrial levels.^45^ Based on our carbonate addition experiments, ongoing ocean acidification is expected to gradually enhance Hg­(II) photoreduction rates if current acidification trends continue. To quantify this effect, *k*_r_ should be parametrized as a function of surface seawater DIC concentrations. Integrating mechanistic dependencies of *k*_*r*_ on DOM composition, salinity, and DIC into global biogeochemical models will significantly enhance our ability to predict Hg cycling and its response to ongoing climate changes.

## Supplementary Material



## Notes

Additional experimental and theoretical details, including preparation of test solutions, measurements of UV-A intensity, experimental determination of *k*_r_ using pseudo-first order and two-step reversible kinetic models, dissolved Hg­(II) speciation obtained using MINEQL+, and cumulative Hg(0) versus time plots (PDF)
