# Supplementary material for: Mechanistic Insights into Mercury Photoreduction: Effects of Dissolved Organic Matter and Inorganic Carbon in Seawater
Source: Environ Sci Technol. 2026 Mar 27;60(14):10840–9. doi: 10.1021/acs.est.5c15961 (PMC13085521; doi:10.1021/acs.est.5c15961)
Supplement: Supplementary file 1 [file es5c15961_si_001.pdf]

Supporting Information

Mechanistic Insights into Mercury Photoreduction: Effects of Dissolved Organic Matter and Inorganic Carbon in Seawater

Sangwoo Eom[1], Huu-Viet Nguyen[1], Asif Qureshi[2], Seunghee Han[1,*]

[1] Department of Environment and Energy Engineering, Gwangju Institute of Science and Technology (GIST), Gwangju 61005, Republic of Korea

[2] Department of Climate Change & Department of Civil Engineering, Indian Institute of Technology (IIT) Hyderabad, Sangareddy, Telangana 502285, India.

* Corresponding author E-mail: shan@gist.ac.kr

This file includes: Supplementary Text S1 to S7; Tables S1 to S8; Figures S1 to S4; References

Text S1. Effects of thiolic DOM on Hg(II) photoreduction.

The $HgCl_2$ solution was equilibrated with thiolic DOM in the dark for 2 to 8 hours,[1] either in DIW, 1 mM PBS solution (pH = 8.0) or in ASW (the Hg–DOM equilibrated solution). We utilized naturally occurring biogenic aliphatic thiols found in the surface seawater, cysteine (CYS), and glutathione (GSH),[2] and a non-biogenic photo-reactive aliphatic thiol, thioglycolic acid (TGA) (Figure S2).[3] Three aromatic thiol molecules, 2-mercaptophenol (2-MP), thiosalicylic acid (TSA), and 4-mercaptobenzoic acid (4-MBA), were additionally used for the Hg(II) photoreduction assays (Figure S2). Photoreduction experiments were conducted with these thiolic DOM at concentrations of 4 and 40 nM, consistent with the thiol-like DOM levels reported in natural seawater (6.9–85 nM),[2] and 2.0 pM Hg(II) prepared with 5 mM $HgCl_2$ stock solution. The final Hg(II) concentration of 2 pM falls within the range of Hg levels reported for surface seawater in the North Western Atlantic Ocean (0.6–4.7 pM)[4] and North Pacific Ocean (0.5–1.9 pM).[5] Under these conditions, the Hg-to-thiol molar ratio in the reaction medium ranges from 5×10^{-5} to 5×10^{-4}, which is comparable to dissolved Hg-to-thiol ratios reported for natural seawater from estuaries and continental shelf regions (3×10^{-5} to 6×10^{-4}).[2,6]

Text S2. Estimation of UV-A intensity using potassium ferrioxalate chemical actinometer. UV-A intensity inside the working solution in the quartz reactor was estimated using potassium ferrioxalate [$K_3Fe(C_2O_4)_3•3H_2O$] chemical actinometers.[7] We mixed 37.8 g of oxalic acid dihydrate ($C_2H_2O_4$) and 33.6 g of potassium hydroxide (KOH) in 80 mL deionized water, which showed the exothermic reaction. After temperature decrease (<60°C), we slowly added 16.2 g of ferric chloride ($FeCl_3$) to produce $K_3Fe(C_2O_4)_3•3H_2O$ crystal under dark condition (Figure S1a). This crystal vigorously rinsed with deionized water and dried for a day. It was finally kept in brown borosilicate amber vials at 4 °C.

The photoreaction mechanisms of $K_3Fe(C_2O_4)_3•3H_2O$ were described as follow. To estimate the photo-produced Fe^{2+} from the potassium ferrioxalate crystal, we plotted a calibration curve using Fe^{2+} standard solution, added with 1,10-phenanthroline (1,10-phen) at pH 5.1 (Figure S1). The slope of the calibration curve was 11,702 M^{-1} cm^{-1}, which aligns with molar absorptivity of $Fe(1,10\text{-phen})_3^{2+}$ (11,100 M^{-1} cm^{-1}) at 510 nm wavelength, indicating that photo-produced Fe^{2+} concentration can be accurately estimated using the calibration curve.

$$[Fe^{III}(C_2O_4)_3]^{3-} + hv \rightarrow [Fe^{II}(C_2O_4)_2]^{2-} + C_2O_4^{-}$$

$$[Fe^{III}(C_2O_4)_3]^{3-} + C_2O_4^{-} \rightarrow [Fe^{III}(C_2O_4)_3]^{2-} + C_2O_4^{2-}$$

$$[Fe^{II}(C_2O_4)_3]^{2-} \rightarrow [Fe^{III}(C_2O_4)_3]^{2-} + 2CO_2$$

For the light intensity measurement, we dissolved 10.3 g of $K_3Fe(C_2O_4)_3•3H_2O$ into 700 mL of 0.1 N H_2SO_4 solution in the quartz reactor to produce 30 mM ferrioxalte solution. We collected 1 mL of aliquots from 700 mL working solution at 0, 5, 9, 15, and 20 minutes under UV-A irradiation (Figure S1b). Then it was added into 49 mL of a buffer solution at pH 5.1, containing 7.5 mL of 1,10-phenanthroline (1,10-phen) to complex Fe^{2+}, as a form of $Fe(1,10\text{-phen})_3^{2+}$ (Figure S1c).

Finally, we measured the absorbance of $Fe^{2+}(1,10\text{-phen})_3{}^{2+}$ to calculate the UV-A intensity inside the quartz reactor. The related calculations are described bellows.

$$I = \Delta n/10^{-3} \cdot \Phi \cdot V_1 \cdot t$$

$$\Delta n = 10^{-3} \cdot V_1 \cdot V_3 \cdot C_t/V_2$$

$$C_t = abs./(\varepsilon \cdot l)$$

where Δn is a mole of photo-generated Fe^{2+}, Φ is a photoreaction quantum yield of Fe^{2+} in the ferrioxalate system (Φ = 1.25 at 280–360 nm wavelength),[8] V_1 is irradiated volume (mL), t is an irradiation time (min), V_2 is the volume taken from the irradiated samples (mL), V_3 is the volume after dilution for concentration determination (mL), C_t is the concentration of Fe^{2+} after dilution, abs is the absorbance of Fe^{2+} at 510 nm wavelength, ε is the molar absorptivity (M^{-1} cm^{-1}) of the $Fe(1,10\text{-phen})_3{}^{2+}$, and l is the light path of the quartz cell. Finally, UV-A intensity inside the quartz reactor was estimated to be 14.6 W m^{-2} in this study.

Text S3. Effects of water matrix and sea salts on Hg(II) photoreduction.

The effect of water matrix on the photoreduction of Hg(II) was evaluated by comparing k_r values of Hg(II) in DIW, 1 mM PBS, 2 mM $NaHCO_3$, 0.4 M NaCl, 2 mM $NaHCO_3$ plus 0.4 mM NaCl, and ASW, in the absence of thiolic DOM, and in 0.4 M NaCl, 0.4 M NaCl plus 2 mM $NaHCO_3$, and ASW, in the presence of 40 nM GSH. Each test solution was spiked with a 5 mM $HgCl_2$ stock solution to achieve a final Hg(II) concentration of 2 pM. To further isolate the role of individual sea salts, k_r of Hg(II) (2 pM) was measured in the presence of 40 nM GSH and each major inorganic salt (Table S1) in the PBS of pH 8.0.

Text S4. Effect of non-thiolic DOM on Hg(II) photoreduction.

We selected anthranilic acid, 4-aminobenzoic acid, salicylic acid, 4-nitrophenol, 2-nitrophenol, and p-benzoquinone as representative aromatic DOMs, as these are not expected to complex with Hg(II) under seawater concentrations of Cl^- (~0.54 M) and thiolic DOM (~40 nM) (Figure S2).[9] The final concentrations of aromatic DOM were set to 400 nM, which represents the upper limit of aromatic DOM levels in coastal seawater (phenolic content: 30–550 nM).[10] We did not prioritize assessing aliphatic DOM in this work because aromatic compounds generally exhibit stronger UV absorption than aliphatic DOM,[11] although certain aliphatic compounds, such as aliphatic amines, can also absorb UV light.[12]

To assess the role of radical species in photoreduction of Hg(II) (2 pM $HgCl_2$) in the presence of 40 nM CYS and 400 nM non-thiolic aromatic DOM molecules, •OH, $^1O_2$, and $^3DOM^*$ were scavenged individually by adding 10 mM 2-propanol, 1 mM furfuryl alcohol, and 0.2 mM sorbic acid, respectively. The same scavenger test was performed with natural seawater collected from the Arctic Ocean after $HgCl_2$ spiking to have a final concentration of 2 pM. The concentrations of these scavengers fall within the recommended ranges for photodegradation studies of MeHg and other organic pollutants.[13]

Text S5. Calculation of k_1, k_{-1}, and k_2 in the two-step reversible kinetic model.

The governing differential equation are described in Eqs. S1 to S3.

$$\frac{d[\mathrm{Hg(II)}]}{dt} = -k_1[\mathrm{Hg(II)}] + k_{-1}[\mathrm{Hg(I)}] \qquad \text{(S1)}$$

$$\frac{d[\mathrm{Hg(I)}]}{dt} = k_1[\mathrm{Hg(II)}] - (k_2 + k_{-1})[\mathrm{Hg(I)}] \qquad \text{(S2)}$$

$$\frac{d[\mathrm{Hg(0)}]}{dt} = k_2[\mathrm{Hg(I)}] \qquad \text{(S3)}$$

The analytical solution for Hg(0) change as a function of time is given by Eq. S4

$$[Hg(0)_t] = [Hg(0)_0]\left[1 - \frac{\lambda_2 e^{-\lambda_1 t} - \lambda_1 e^{-\lambda_2 t}}{\lambda_2 - \lambda_1}\right] \qquad \text{(S4)}$$

where, λ_1 *and* λ_2 are the characteristic rate coefficients defined as Eqs. S5 and S6.

$$\lambda_1 = \frac{(k_1 + k_{-1} + k_2) + \sqrt{(k_1 + k_{-1} + k_2)^2 - 4k_1k_2}}{2} \qquad \text{(S5)}$$

$$\lambda_2 = \frac{(k_1 + k_{-1} + k_2) - \sqrt{(k_1 + k_{-1} + k_2)^2 - 4k_1k_2}}{2} \qquad \text{(S6)}$$

Initially, k_1 and k_2 were estimated by two-step one electron transfer model fitting to the curve of Hg(0) versus time obtained from the scavenger reactions, assuming $k_{-1} = 0$ (Eq. S7).

$$\mathrm{Hg(II)} \underset{k_1}{\rightarrow} \mathrm{Hg(I)} \underset{k_2}{\rightarrow} \mathrm{Hg(0)} \qquad \text{(S7)}$$

This approach isolates the forward reduction from the reverse oxidation step, since the added scavengers are assumed to remove all the intermediate oxidants, such as ^{3}DOM*, •OH, and $^1O_2$.[14] The rate equations can be expressed as described in Eqs. S8 to S10.

$$\frac{d[\mathrm{Hg(II)}]}{dt} = -k_1[\mathrm{Hg(II)}] \qquad \text{(S8)}$$

$$\frac{d[\mathrm{Hg(I)}]}{dt} = k_1[\mathrm{Hg(II)}] - k_2[\mathrm{Hg(I)}] \qquad \text{(S9)}$$

$$\frac{d[\mathrm{Hg(0)}]}{dt} = k_2[\mathrm{Hg(I)}] \qquad \text{(S10)}$$

The analytical solution for the evolution of $[\mathrm{Hg(0)}]$ is given by Eq. S11.

$$[Hg(0)_t] = [Hg(0)_0]\left[1 - \frac{k_2 e^{-k_1 t} - k_1 e^{-k_2 t}}{k_2 - k_1}\right] \quad \text{(S11)}$$

Once k_1 and k_2 are determined, k_{-1} can be estimated from the control reactions carried out without scavenger addition using Eqs. S1 to S6, assuming k_1 and k_2 are same as those obtained from the scavenger reaction.

Table S1. Sea salt composition in artificial seawater.[15]

Inorganic salt	Concentration (g L^{-1})	Concentration (M)
$NaCl$	23.960	0.41
Na_2SO_4	3.977	0.028
$MgCl_2$	5.046	0.053
$CaCl_2$	1.110	0.010
KCl	0.678	0.0091
$NaHCO_3$	0.193	0.0023
KBr	0.098	0.00082
$B(OH)_3$	0.025	0.00040
NaF	0.003	0.000071

Table S2 Changes in pH, total alkalinity, and dissolved inorganic carbon in various matrices under initial conditions and after 12 h of aeration. Total alkalinity and dissolved inorganic carbon were measured using potentiometric titration and coulometric analysis, respectively.[16]

Matrix	Aeration time	pH	Total alkalinity (μmol kg^{-1})	Dissolved inorganic carbon (μmol kg^{-1})	Dissolved oxygen (mg L^{-1})
DIW	0 h	7.0	85	17	7.8
	12 h	6.9	83	23	8.5
PBS	0 h	8.0	994	30	7.6
	12 h	7.4	974	270	8.5
ASW	0 h	7.4	2217	2203	5.8
	12 h	7.8	2259	2090	8.8
NSW	0 h	8.2	1230	894	-
	12 h	7.7	1044	1007	-

Table S3. The experimentally determined k_r and R^2 values denoting goodness of fit of pseudo-first order kinetic model. The k_r values are described in Figure 1.

Matrix	Thiol concentration (nM)	Thiol type	k_r (h^{-1})	R^2
DIW	4	2-MP	1.3	0.98
			0.87	0.97
		TSA	0.59	0.99
			0.54	1.0
		4-MBA	0.80	0.99
			1.1	0.99
	40	2-MP	1.0	0.99
			1.2	0.95
		TSA	0.65	0.99
			0.64	0.98
		4-MBA	0.87	0.99
			0.84	0.98
PBS	4	2-MP	0.41	1.0
			0.52	0.98
		TSA	0.51	0.98
			0.49	0.99
		4-MBA	1.3	0.99
			1.2	1.0
	40	2-MP	0.62	0.97
			0.59	0.98
		TSA	0.56	0.98
			0.52	0.98
		4-MBA	0.81	0.99
			0.96	0.99

Table S3. (continued)

Matrix	Thiol concentration (nM)	Thiol type	k_r (h^{-1})	R^2
ASW	4	CYS	0.05	1.0
			0.11	0.99
		GSH	0.10	0.99
			0.16	0.96
		TGA	0.18	0.94
			0.30	1.0
		TSA	0.29	0.93
			0.20	0.99
		4-MBA	0.52	0.74
			0.73	0.82
	40	CYS	0.16	0.99
			0.25	0.99
			0.23	0.99
		GSH	0.37	1.0
			0.39	1.0
			0.35	1.0
		TGA	0.45	0.99
			0.56	1.0
			0.50	0.99
		2-MP	0.08	1.0
			0.08	1.0
		TSA	0.49	0.97
			0.55	0.98
		4-MBA	1.4	1.0
			1.6	0.99

Table S4. Dissolved Hg(II) species in percentage of total Hg and k_r value obtained in each solution. Hg(II) concentration is 2 pM, ligand concentration is 4 or 40 nM in DI water (DIW), phosphate buffer solution (PBS), and artificial seawater (ASW) prepared as described in Table S1. N.D. denotes not detected. Major Hg(II) species are highlighted as bold. The k_r values are organized in Figure 1. Note that the stability constants (Log K_s) in this table are reported relative to $Hg(OH)_2$, as used in MINEQL+ v5.0.

Type	Ligand	Hg species	Log K_s	DIW (4 nM thiol)	DIW (40 nM thiol)	PBS (4 nM thiol)	PBS (40 nM thiol)	ASW (4 nM thiol)	ASW (40 nM thiol)
Aliphatic thiols	CYS	Hg(CYS)	41.9	7.3	7.2	42	35	35	30
		**$Hg(CYS)H^+$**	50.0	**92**	**90**	**56**	**47**	**63**	**54**
		$Hg(CYS)H_2^{2+}$	52.3	0.0018	0.0018	0.00013	0.00011	0.00034	0.00029
		$Hg(CYS)_2^{2-}$	50.7	0	0.00077	0.043	0.36	0.10	0.87
		$Hg(CYS)_2H^-$	60.2	0.021	0.20	0.97	8.2	0.95	8.2
		$Hg(CYS)_2H_2$	68.2	0.24	2.4	1.1	9.1	0.80	6.8
		k_r (h^{-1})	-	N.D.	N.D.	N.D.	N.D.	0.081	0.21
	GSH	$Hg(GSH)^-$	39.7	8.1	1.1	11	1.2	7.3	0.80
		Hg(GSH)H	47.2	23	3.3	3.0	0.34	1.5	0.16
		$Hg(GSH)H_2^+$	50.4	0.014	0.0020	0.00019	0	0.00013	0
		$Hg(GSH)_2^{4-}$	47.5	0.00012	0.00017	0.028	0.032	0.83	0.90
		$Hg(GSH)_2H^{3-}$	51.0	0.25	0.35	3.9	4.5	15	16
		**$Hg(GSH)_2H_2^{2-}$**	67.3	**68**	**95**	**82**	**94**	**75**	**82**
		$Hg(GSH)_2H_3^-$	71.8	0.22	0.31	0.023	0.026	0.0087	0.0095
		k_r (h^{-1})	-	N.D.	N.D.	N.D.	N.D.	0.13	0.37

Table S4. ***(Continued)***

Type	Ligand	Hg species	Log K_s	DIW (4 nM thiol)	DIW (40 nM thiol)	PBS (4 nM thiol)	PBS (40 nM thiol)	ASW (4 nM thiol)	ASW (40 nM thiol)
Aliphatic thiols	TGA	**Hg(TGA)**	41.1	**99**	**92**	**91**	50	**81**	30
		**$Hg(TGA)_2^{2-}$**	50.4	0.87	8.1	9.2	**50**	19	**70**
		k_r (h^{-1})	-	N.D.	N.D.	N.D.	N.D.	0.24	0.50
Aromatic thiols	TSA	$HgCl_2$	20.2	0	0	0	0	0.058	0.0010
		$HgCl_3^{--}$	21.2	0	0	0	0	0.32	0.0056
		$HgCl_4^{2-}$	21.8	0	0	0	0	1.4	0.024
		**Hg(TSA)**	31.5	**91**	49	**57**	12	46	8.2
		**$Hg(TSA)_2$**	40.1	9.4	**51**	43	**88**	**52**	**92**
		k_r (h^{-1})	-	0.57	0.64	0.50	0.54	0.25	0.52

Table S5. These are experimentally determined k_r values and R^2 denoting goodness of fit of pseudo-first order kinetic model. The k_r values are described in Figures 3 and S4.

Matrix	Thiol type	k_r (h^{-1})	R^2
DIW	-	0.23	0.99
		0.32	0.97
PBS	-	0.27	1.0
		0.38	0.99
2 mM $NaHCO_3$	-	0.20	1.0
		0.24	0.99
0.4 M NaCl + 2 mM $NaHCO_3$	40 nM GSH	0.38	1.0
		0.36	1.0
ASW	40 nM GSH	0.37	1.0
		0.39	1.0
		0.35	1.0
ASW without $NaHCO_3$ + 1 mM $NaHCO_3$	40 nM GSH	0.23	1.0
ASW without $NaHCO_3$ + 0.2 mM $NaHCO_3$	40 nM GSH	1.8×10^{-7}	0.93
0.7 M NaCl + 2 mM $NaHCO_3$	40 nM GSH	2.1×10^{-7}	0.99
Natural seawater	-	0.28	1.0

Table S6. Dissolved Hg(II) species in percentage of total Hg(II) and k_r value obtained in each solution. Hg(II) concentration is 2.0 pM and GSH concentration is 40 nM. Note that the stability constants (Log K_s) in this table are reported relative to $Hg(OH)_2$, as used in MINEQL+ v5.0.

Hg species	Log K_s	Without GSH (%)						With GSH (%)			
		DIW	PBS	2 mM $NaHCO_3$	0.4 M NaCl	0.4 M NaCl + 2 mM $NaHCO_3$	ASW	DIW	0.4 M NaCl	0.4 M NaCl + 2 mM $NaHCO_3$	ASW
**$Hg(OH)_2$**	6.19	**100**	**100**	**100**	0	0	0.0014	0	0	0	0
HgClOH	10.4	0	0	0	0.033	0	0.15	0	0	0	0
$HgCl_2$	20.2	0	0	0	5.4	5.4	3.3	0	0	0	0
$HgCl_3^-$	21.2	0	0	0	22	22	18	0	0	0	0
**$HgCl_4^{2-}$**	21.8	0	0	0	**72**	**72**	**78**	0	0	0	0
$Hg(GSH)^-$	39.7	-	-	-	-	-	-	1.1	0.60	0.80	0.80
Hg(GSH)H	47.2	-	-	-	-	-	-	3.3	1.2	0.16	0.16
$Hg(GSH)H_2^+$	50.4	-	-	-	-	-	-	0.0020	0.00010	0	0
$Hg(GSH)_2^{4-}$	47.5	-	-	-	-	-	-	0.00017	0.011	0.92	0.90
$Hg(GSH)_2H^{3-}$	51.0	-	-	-	-	-	-	0.35	2.0	17	16
**$Hg(GSH)_2H_2^{2-}$**	67.3	-	-	-	-	-	-	**95**	**96**	**82**	**82**
$Hg(GSH)_2H_3^-$	71.8	-	-	-	-	-	-	0.31	0.11	0.0094	0.0095
k_r (h^{-1})	-	0.28	0.34	0.22	N.D.	N.D.	N.D.	N.D.	N.D.	0.37	0.37

Table S7. The experimentally determined k_r values and R^2 values denoting goodness of fit of the pseudo-first order kinetic model. The k_r values are described in Figures 4 and 5.

Matrix	Thiol type	Non-thiolic aromatic OM	Scavenger	k_r (h^{-1})	R^2
ASW	CYS	-	-	0.16	0.99
				0.25	0.99
				0.23	0.99
			2-propanol	0.32	0.99
			furfuryl alcohol	0.46	1.0
			sorbic acid	0.45	0.99
		anthranilic acid	-	0.26	0.96
				0.23	0.98
		4-aminobenzoic acid	-	0.18	1.0
				0.18	1.0
			2-propanol	0.38	0.99
			furfuryl alcohol	0.48	1.0
			sorbic acid	0.51	1.0
		salicylic acid	-	0.14	1.0
				0.12	1.0
			2-propanol	0.47	0.99
			furfuryl alcohol	0.50	1.0
			sorbic acid	0.47	1.0
		4-nitrophenol	-	0.24	0.99
				0.18	0.99
		2-nitrophenol	-	0.13	0.99
				0.10	1.0
		p-benzoquinone	-	0.14	1.0
				0.16	0.99
ASW	GSH	-	-	0.37	1.0
				0.39	1.0
				0.35	1.0
			2-propanol	0.51	1.0
			furfuryl alcohol	0.47	1.0
			sorbic acid	0.45	1.0
Natural seawater	-	-	-	0.28	1.0
			2-propanol	0.29	1.0
			furfuryl alcohol	0.33	1.0
			sorbic acid	0.29	1.0

Table S8. Sensitivity analyses were performed using k_1, k_2, and k_{-1} values obtained under conditions of 2 pM Hg(II) and 40 nM CYS in ASW to evaluate changes in k_{-1} resulting from ±10% variations in k_1 and k_2.

	Condition	Two-step reversible model			
		k_1	k_2	k_{-1}	R^2
	Control	0.88	4.4	14	0.98
	$k_1 + 0.1\ k_1$	0.97	4.4	16	0.98
	$k_1 - 0.1\ k_1$	0.79	4.4	12	0.98
	$k_2 + 0.1\ k_2$	0.88	4.8	15	0.98
	$k_2 - 0.1\ k_2$	0.88	4.0	12	0.98
	Control	0.88	4.4	7.6	0.97
	$k_1 + 0.1\ k_1$	0.97	4.4	8.8	0.97
CYS in ASW	$k_1 - 0.1\ k_1$	0.79	4.4	6.4	0.97
	$k_2 + 0.1\ k_2$	0.88	4.8	8.5	0.97
	$k_2 - 0.1\ k_2$	0.88	4.0	6.8	0.97
	Control	0.88	4.4	12	0.96
	$k_1 + 0.1\ k_1$	0.97	4.4	14	0.95
	$k_1 - 0.1\ k_1$	0.79	4.4	10	0.97
	$k_2 + 0.1\ k_2$	0.88	4.8	14	0.95
	$k_2 - 0.1\ k_2$	0.88	4.0	11	0.97

Figure S1. a) Potassium ferrioxalate ($K_3Fe(C_2O_4)_3 \cdot 3H_2O$) chemical actinometers in an Erlenmeyer flask; b) UV-A incubator to photochemically produce Fe^{2+} in the working solution contained in the quartz reactor; c) $Fe(1,10\text{-phen})_3^{2+}$ in the buffer solution at pH 5.1 incubated under the UV-A irradiation; d) absorbance at 510 nm versus Fe^{2+} concentration; and e) absorbance at 510 nm of photo-produced Fe^{2+} versus time.

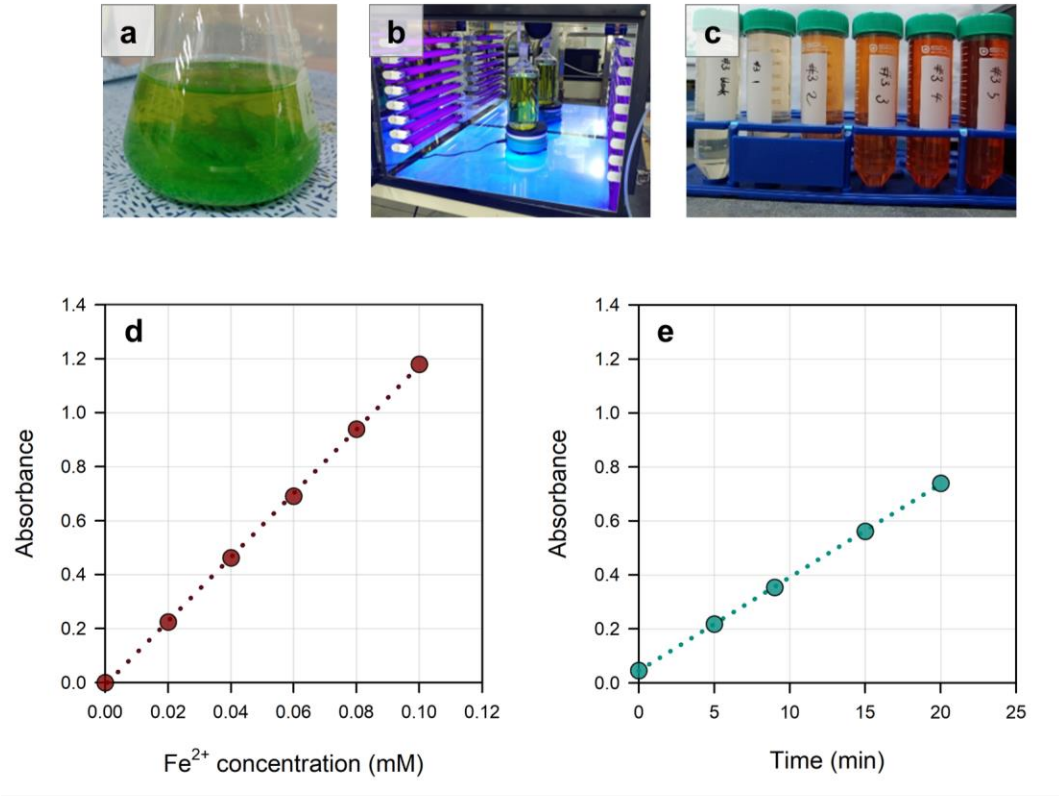

Figure S2. Molecular structures of three aliphatic thiols, three aromatic thiols, and six non-thiolic aromatic DOM used for Hg(II) photoreduction tests in this study.

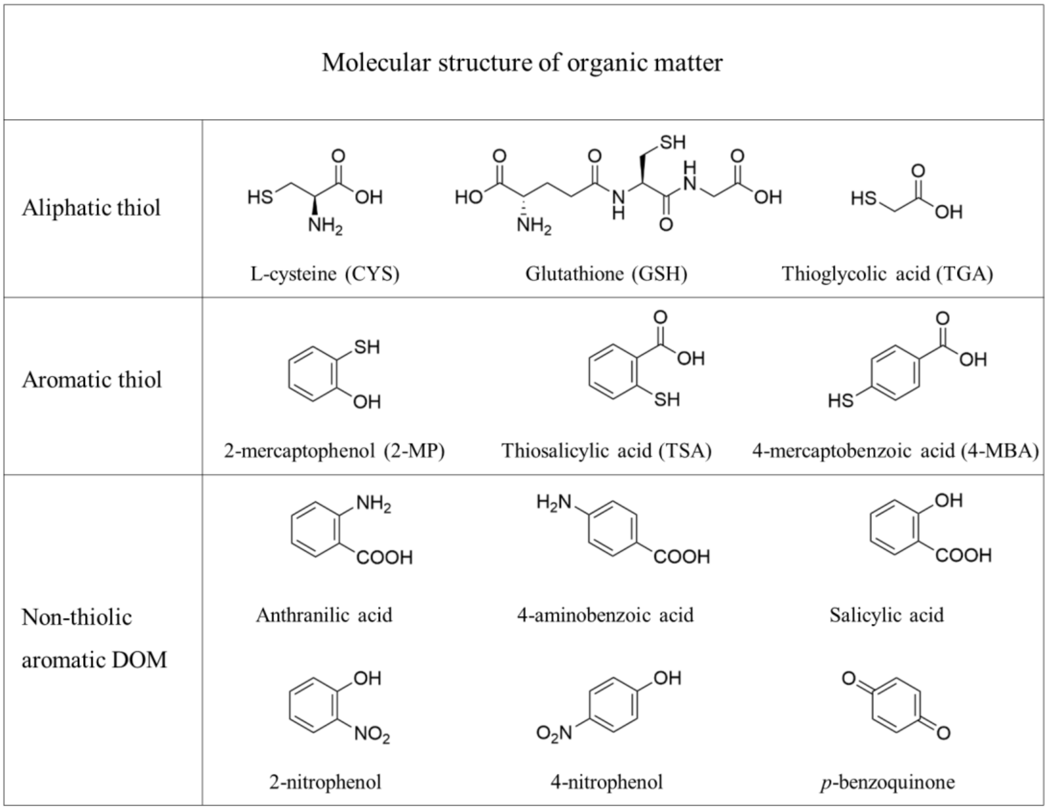

Figure S3. The concentration effect of (a) bicarbonate and (b) NaCl on the cumulative Hg(0) production under UV-A in the presence of 2 pM Hg(II) and 40 nM glutathione (GSH). (c) Cumulative Hg(0) production under UV-A in the presence of 2 pM Hg(II) dissolved in natural seawater (NSW) collected from the Arctic Ocean. Reaction medium for (a) is artificial seawater prepared without $NaHCO_3$.

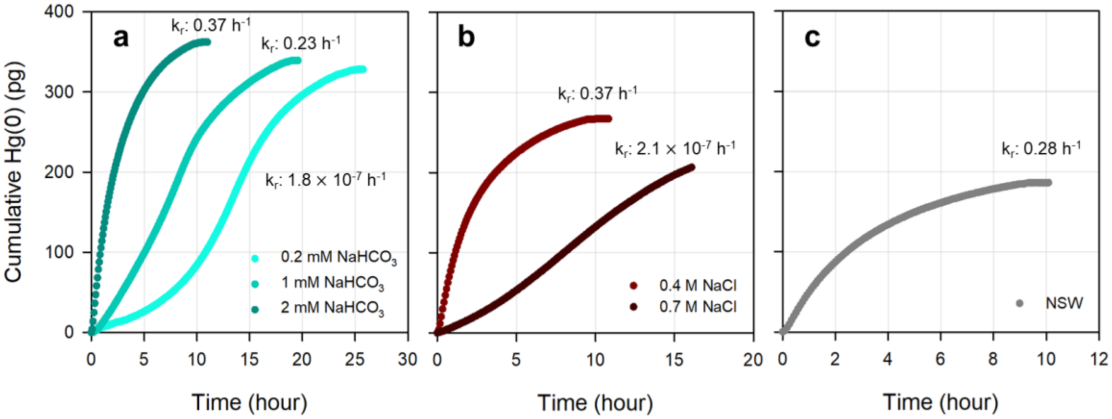

REFERENCES

(1) Wiederhold, J. G.; Cramer, C. J.; Daniel, K.; Infante, I.; Bourdon, B.; Kretzschmar, R. Equilibrium mercury isotope fractionation between dissolved Hg (II) species and thiol-bound Hg. *Environmental science & technology* **2010**, *44*, 4191-4197. Wang, Q.; Zhang, L.; Liang, X.; Yin, X.; Zhang, Y.; Zheng, W.; Pierce, E. M.; Gu, B. Rates and dynamics of mercury isotope exchange between dissolved elemental Hg (0) and Hg (II) bound to organic and inorganic ligands. *Environmental Science & Technology* **2020**, *54*, 15534-15545.

(2) Gao, Z.; Guéguen, C. Distribution of thiol, humic substances and colored dissolved organic matter during the 2015 Canadian Arctic GEOTRACES cruises. *Marine Chemistry* **2018**, *203*, 1-9.

(3) Jeremiason, J. D.; Portner, J. C.; Aiken, G. R.; Hiranaka, A. J.; Dvorak, M. T.; Tran, K. T.; Latch, D. E. Photoreduction of Hg (II) and photodemethylation of methylmercury: the key role of thiol sites on dissolved organic matter. *Environmental Science: Processes & Impacts* **2015**, *17*, 1892-1903.

(4) Soerensen, A. L.; Mason, R. P.; Balcom, P. H.; Sunderland, E. M. Drivers of surface ocean mercury concentrations and air–sea exchange in the West Atlantic Ocean. *Environmental science & technology* **2013**, *47*, 7757-7765.

(5) Sunderland, E. M.; Krabbenhoft, D. P.; Moreau, J. W.; Strode, S. A.; Landing, W. M. Mercury sources, distribution, and bioavailability in the North Pacific Ocean: Insights from data and models. *Global Biogeochemical Cycles* **2009**, *23*.

(6) Seelen, E.; Liem-Nguyen, V.; Wünsch, U.; Baumann, Z.; Mason, R.; Skyllberg, U.; Björn, E. Dissolved organic matter thiol concentrations determine methylmercury bioavailability across the terrestrial-marine aquatic continuum. *Nature Communications* **2023**, *14*, 6728. Kirk, J. L.; St. Louis, V. L.; Hintelmann, H.; Lehnherr, I.; Else, B.; Poissant, L. Methylated mercury species in marine waters of the Canadian high and sub Arctic. *Environmental science & technology* **2008**, *42*, 8367-8373.

(7) Hatchard, C.; Parker, C. A. A new sensitive chemical actinometer-II. Potassium ferrioxalate as a standard chemical actinometer. *Proceedings of the Royal Society of London. Series A. Mathematical and Physical Sciences* **1956**, *235*, 518-536.

(8) Goldstein, S.; Rabani, J. The ferrioxalate and iodide–iodate actinometers in the UV region. *Journal of Photochemistry and Photobiology A: Chemistry* **2008**, *193*, 50-55.

(9) He, F.; Zheng, W.; Liang, L.; Gu, B. Mercury photolytic transformation affected by low-molecular-weight natural organics in water. *Science of the total environment* **2012**, *416*, 429-435. Luo, H.-W.; Yin, X.; Jubb, A. M.; Chen, H.; Lu, X.; Zhang, W.; Lin, H.; Yu, H.-Q.; Liang, L.; Sheng, G.-P. Photochemical reactions between mercury (Hg) and dissolved organic matter decrease Hg bioavailability and methylation. *Environmental Pollution* **2017**, *220*, 1359-1365.

(10) Takeda, K.; Moriki, M.; Oshiro, W.; Sakugawa, H. Determination of phenolic concentrations in dissolved organic matter pre-concentrate using solid phase extraction from natural water. *Marine Chemistry* **2013**, *157*, 208-215.

(11) He, F.; Zhao, W.; Liang, L.; Gu, B. Photochemical oxidation of dissolved elemental mercury by carbonate radicals in water. *Environmental Science & Technology Letters* **2014**, *1*, 499-503. Turro, N.; Ramamurthy, V.; Cherry, W.; Farneth, W. The effect of wavelength on organic photoreactions in solution. Reactions from upper excited states. *Chemical Reviews* **1978**, *78*, 125-145.

(12) Ishida, H.; Tsubomura, H. Absorption spectra arising from the interaction between aromatic hydrocarbons and aliphatic amines. *Chemical Physics Letters* **1971**, *9*, 296-298.

(13) Carlos, L.; Martire, D. O.; Gonzalez, M. C.; Gomis, J.; Bernabeu, A.; Amat, A. M.; Arques, A. Photochemical fate of a mixture of emerging pollutants in the presence of humic substances. *Water Research* **2012**, *46*, 4732-4740. Han, X.; Li, Y.; Li, D.; Liu, C. Role of free radicals/reactive oxygen species in MeHg photodegradation: Importance of utilizing appropriate scavengers. *Environmental Science & Technology* **2017**, *51*, 3784-3793.
(14) Elsner, M. Stable isotope fractionation to investigate natural transformation mechanisms of organic contaminants: principles, prospects and limitations. *Journal of Environmental Monitoring* **2010**, *12*, 2005-2031.
(15) Kester, D. R.; Duedall, I. W.; Connors, D. N.; Pytkowicz, R. M. Preparation of artificial seawater 1. *Limnology and oceanography* **1967**, *12*, 176-179.
(16) Lee, C.-H.; Subhas, A. V.; Kim, J.-H.; Lee, K. Ocean Carbon Dioxide Removal and Storage. *Chemical Reviews* **2026**.
